# Hydrogen Bonding in the Dimer and Monohydrate of 2-Adamantanol: A Test Case for Dispersion-Corrected Density Functional Methods

**DOI:** 10.3390/molecules27082584

**Published:** 2022-04-17

**Authors:** Marcos Juanes, Rizalina Tama Saragi, Cristóbal Pérez, Luca Evangelisti, Lourdes Enríquez, Martín Jaraíz, Alberto Lesarri

**Affiliations:** 1 Departamento de Química Física y Química Inorgánica, Facultad de Ciencias—I.U. CINQUIMA, Universidad de Valladolid, Paseo de Belén, 7, 47011 Valladolid, Spain; marcos.juanes@uva.es (M.J.); rizalinatama.saragi@uva.es (R.T.S.); cristobal.perez@uva.es (C.P.); 2 Dipartimento di Chimica ‘‘Giacomo Ciamician’’, Università di Bologna, Via Selmi, 2, 40126 Bologna, Italy; luca.evangelisti6@unibo.it; 3 Departamento de Electrónica, Escuela Técnica Superior de Ingenieros de Telecomunicación, Universidad de Valladolid, Paseo de Belén, 15, 47011 Valladolid, Spain; louenr@tel.uva.es (L.E.); mjaraiz@ele.uva.es (M.J.)

**Keywords:** chiral recognition, transient chirality, non-covalent interactions, hydrogen bonding, rotational spectroscopy, jet spectroscopy

## Abstract

Weakly-bound intermolecular clusters constitute reductionist physical models for non-covalent interactions. Here we report the observation of the monomer, the dimer and the monohydrate of 2-adamantanol, a secondary alcohol with a bulky ten-carbon aliphatic skeleton. The molecular species were generated in a supersonic jet expansion and characterized using broadband chirped-pulse microwave spectroscopy in the 2–8 GHz frequency region. Two different *gauche*-*gauche* O-H···O hydrogen-bonded isomers were observed for the dimer of 2-adamantanol, while a single isomer was observed for the monomer and the monohydrate. The experimental rotational parameters were compared with molecular orbital calculations using density functional theory (B3LYP-D3(BJ), B2PLYP-D3(BJ), CAM-B3LYP-D3(BJ), ωB97XD), additionally providing energetic and electron density characterization. The shallow potential energy surface makes the dimer an interesting case study to benchmark dispersion-corrected computational methods and conformational search procedures.

## 1. Introduction

The formation of intermolecular clusters in the gas phase, mostly through jet-cooled supersonic expansions, has been used since the 1980s as a major experimental tool for the evaluation of the structural and energetic factors controlling non-covalent interactions (NCI) [1,2], in particular the hydrogen bond [3,4]. The investigation of gas-phase clusters ideally combines with first-principles electronic structure calculations [5,6,7], providing structural descriptions unperturbed by the matrix effects present in condensed phases. Electronic, vibrational and rotational spectroscopy all contribute experimental information on NCI, but they differ in the molecular size range and structural content. 

Electronic and vibrational spectroscopy is appropriate for larger clusters such as trimers to decamers. Moreover, when combined with IR/UV double-resonance ion-dip laser techniques, it efficiently provides mass and conformer selection [8]. However, structural identification is not always unequivocal from pure vibronic data. Conversely, rotational spectroscopy delivers direct structural characterization of NCI through the moments of inertia but is limited to small clusters (generally dimer-trimers below 600 Da) [9,10,11].

In this context, the hydration and dimerization of alcohols have been used as molecular probes for non-covalent interactions, molecular recognition and transient chirality involving the canonical O-H···O hydrogen bond. The microsolvation of alcohols permits observing the amphoteric character of water [12], preferentially acting as a proton donor to aliphatic alcohols [13,14,15,16] but as a proton acceptor in aromatic molecules such as phenol [17] or propofol [18], or in fluorinated aliphatics [19]. 

We recently explored the hydration [20] and dimerization [21] of the six-membered ring of cyclohexanol. The rationale for this study was the comparison with aromatic alcohols, examining the differences with benzyl, furfuryl or thenyl dimers and hydrates, such as those of phenol [22], benzyl alcohol [23,24], furfuryl alcohol [25,26] or thenyl alcohol [26,27]. While all alcohol dimers are primary bound by the O-H···O hydrogen bond, the use of saturated aliphatic rings effectively cancels all intermolecular interactions associated to the π-ring systems, such as O-H···π, C-H···π or π···π, offering a different balance of secondary interactions and dispersion contributions. The presence of a single leading interaction in the cyclohexanol dimer produced a rich conformational landscape for the complex, with six competing isomers. At the same time, the lack of relevant secondary interactions makes the potential energy surface quite shallow and corrugated, and our conformational search encountered trouble with finding all the experimental minima. For these reasons, these kinds of dimers can be used as test systems to improve the performance of dispersion-corrected density functional models and conformational search methods.

**Scheme 1:**
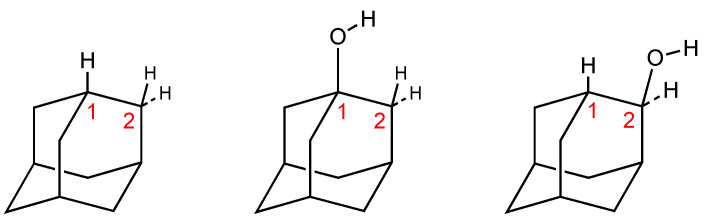
Adamantane (**left**), 1-adamantanol (**center**) and 2-adamantanol (**right**).

In this work, we decided to extend our studies to the dimer of 2-adamantanol (tricyclo [3.3.1.1^3,7^] decan-2-ol, Scheme 1). Adamantane is a tricyclic alkane with a highly-symmetric *T_d_* diamondoid structure and only two symmetry-distinct (methylene or methine) carbon sites. The inclusion of a hydroxyl group may thus produce either a tertiary or secondary alcohol. We chose 2-adamantanol to obtain a secondary alcohol as in cyclohexanol but with a larger 10-carbon skeleton. The larger positive inductive effect and bulky side chain in adamantanol may affect the intermolecular interactions in the dimer and offer a comparison with the potential energy surface (PES) of cyclohexanol. 

2-adamantanol shows asymmetric units of three molecules in low-temperature crystal structures, forming clusters of six hydrogen-bonded molecules [28]. However, there are no previous high-resolution spectroscopic studies in the gas phase. A Stark modulation microwave spectrum of 1-adamantanol calculated a barrier of 4.9(4) kJ mol^−1^ [29] for the internal rotation of the alcohol group, in line with other alcohols. The jet-cooled FT-IR spectrum of 2-adamantanol by Suhm [30] reported OH stretching bands for the monomer (3650 cm^−1^) and dimer (3520 cm^−1^), but the single red-shifted band of the dimer did not permit verification of the presence of different isomers. Solid-state FT-IR spectra are available for the 1-adamantanol monomer [31].

## 2. Materials and Methods

Similarly to adamantane, 2-adamantanol is a solid with a high melting point (300 °C). However, it was expected that vaporization could be possible by sublimation [32]. To this purpose, a commercial sample of 2-adamantanol (97%) was inserted in a heating reservoir of a pulsed (solenoid-driven) gas injector, backed by a stream of an inert carrier gas (neon at stagnation pressures of 0.2 MPa bar). A mild temperature (<50–70 °C) finally proved acceptable to obtain the spectrum. For the observation of the water dimer, a liquid reservoir was inserted in the carrier gas line. The gas mixture formed a supersonic jet by near-adiabatic expansion through a single nozzle (diameter of 0.5 or 0.8 mm), using typical gas pulses of 800–900 μs. The jet was probed in the 2–8 GHz cm-wave region with a chirped-pulse Fourier transform microwave (CP-FTMW) spectrometer, following a direct-digital design by Pate [33,34]. Sample excitation was achieved by sequences of short (4 μs, 20 W) chirped pulses, broadcast perpendicularly to the jet. For each experimental cycle, the MW radiation induces a fast-passage transient excitation of the polar molecules, covering the full spectral bandwidth [35,36]. The molecular ensemble subsequently emits a free-induction decay, which is detected in the time domain (ca. 40 μs) and acquired using a 25 GSamples/s digital oscilloscope. A Fourier transformation yields the frequency-domain spectrum. A Kaiser–Bessel window is used for apodization, producing spectral FWHM linewidths of about 100 kHz. In the present experiment, 1 M cycles were averaged at a repetition rate of 5 Hz. The uncertainty of the frequency measurements is estimated to be better than 20 kHz. 

Several computational methods were used to rationalize the experimental results. A blind conformational search using molecular mechanics (MMFFs [37]) produced an initial set of plausible structures, which were reoptimized using molecular orbital methods. Based on our previous experience, two computationally-effective density functional methods (DFT) were first selected, including the hybrid B3LYP [38] and double-hybrid B2PLYP [39] functionals, which were combined with Ahlrichs’ polarized triple-zeta basis set def2-TZVP [40]. In both cases, the calculations were supplemented with Grimme’s D3 [41] dispersion corrections and Becke-Johnson damping function [42], which generally proved effective in previous spectroscopic studies [21]. In a later stage, the most stable 2-adamantanol dimer geometries were also reoptimized using the long-range corrected Coulomb-attenuating method CAM-B3LYP-D3(BJ) [43] and the long-range atom–atom dispersion-corrected method ωB97XD [44] and the same basis set. The frequency calculations used the harmonic approximation and the same levels of theory. The complexation energies were calculated taking into account the basis set superposition errors (BSSE) with the counterpoise approximation [7]. All DFT calculations were implemented in Gaussian16 [45].

For the calculation of interconversion barriers between isomers of the dimer of 2-adamantanol, the intermediates, products and transition states were located by means of the GRRM (Global Reaction Route Mapping) program [46], linked to Gaussian16. Transition state structures were optimized as saddle points at the B3LYP-D3(BJ)/6-31g(d) level of calculation. To verify that they connect the expected reactant and product wells, intrinsic reaction coordinate (IRC) calculations were performed at the same level. The found minima and transition state geometries were then reoptimized at the B3LYP-D3(BJ)/def2-TZVP level of theory. 

The physical contributions to the binding potential of the water clusters were analysed using energy decomposition methods and symmetry-adapted perturbation theory (SAPT) [7,47], using Psi4 [48]. Finally, a topological analysis of the electron density was used to assess the presence of non-covalent interactions, using Johnson–Contreras NCIPlot reduced electron density method [49]. 

## 3. Results

### 3.1. 2-Adamantanol Monomer

**Figure 1:**
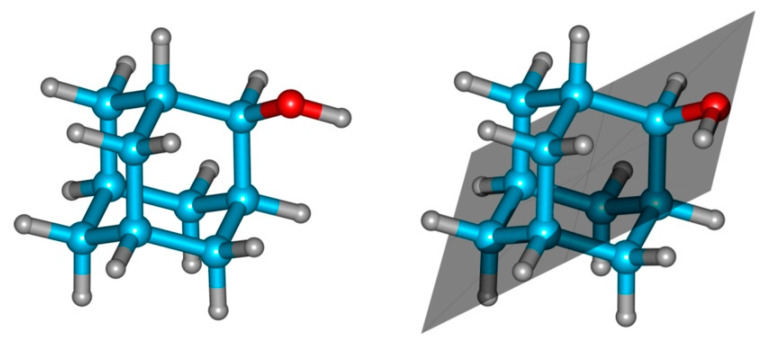
The *gauche* (**left**, global minimum) and *C_s_* plane-symmetric *anti* (**right**) conformations of 2-adamantanol.

The monomer of 2-adamantanol contains a rigid sidechain attached to the torsionally labile hydroxyl group. The hydroxyl group is expected to adopt, preferentially, a staggered position with respect to the vicinal *ipso* hydrogen atom, producing either one *antiperiplanar* (later *anti*) or two equivalent *synclinal* (later *gauche*) conformations with respect to the methine (C-H) hydrogen atom. The computational predictions in Table 1 (B2PLYP) and Table S1 (B3LYP) in the Supplementary Materials (SM) suggested larger stability for the *gauche* isomer of Figure 1, with the *anti* structure at a relative Gibbs energy of 1.8–2.5 kJ mol^−1^. The rotational spectrum revealed a single near-prolate asymmetric rotor, mostly composed of R- (*J*+1←*J*) and Q-branch (*J*←*J*) *μ*_b_ transitions. A small number of ^a^R and ^c^Q transitions were also detected. We observed no indications of torsional tunnelling effects, so the observed transitions were fitted to the Watson’s (S-reduced) semirigid-rotor Hamiltonian, including quartic centrifugal distortion terms [50]. The monosubstituted ^13^C species in natural abundance (1.1%) were detectable, but they will be reported elsewhere. The experimental results are also presented in Table 1 and Table S1 (SM) for comparison with the theory, with the fitted transitions collected in Table S2 (SM). Because of the structural similarities between the *gauche* and *anti* conformations, the predicted inertial parameters are quite close. However, the observed intensities of the *μ*_c_ (absent for the *anti* form) and *μ*_a_ transitions clearly identified the carrier of the spectrum as the *gauche* form, consistent with the predictions for the global minimum. Interestingly, the B3LYP calculations produce a slightly better agreement with the experiment than B2PLYP (relative differences with the rotational constants below 0.5%).

### 3.2. 2-Adamantanol Monohydrate

**Figure 2:**
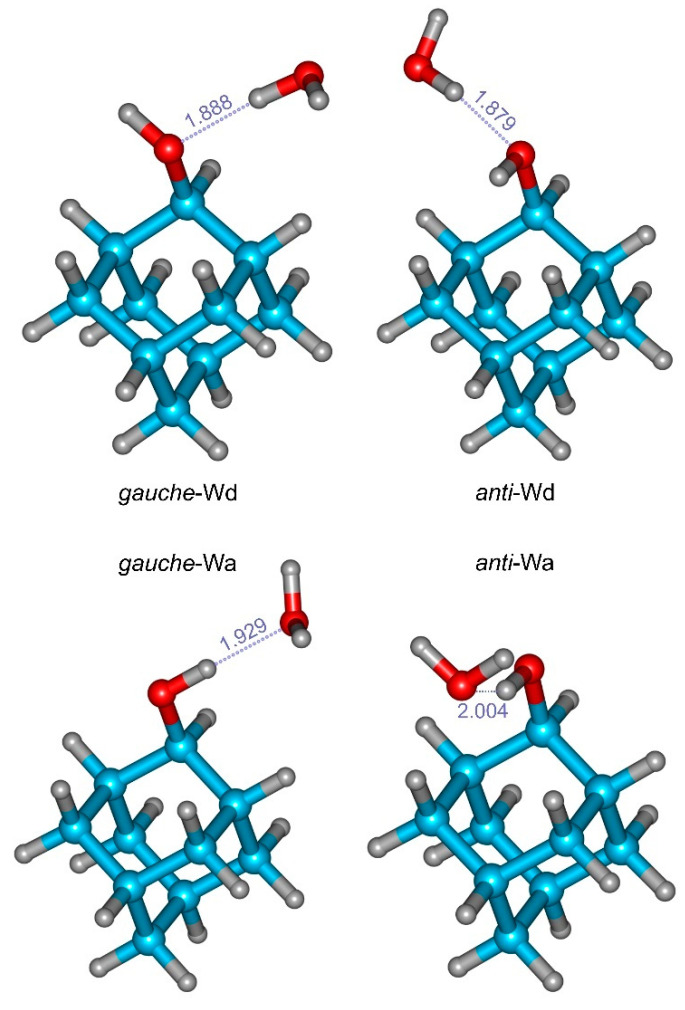
The water donor (Wd, first row) and water acceptor (Wa, second row) isomers of the 2-adamantanol-water monohydrate. The *gauche* and *anti* isomers are shown in the left and right columns, respectively. The O-H···O hydrogen bond is represented by a dotted line, together with the bonding distances predicted with B3LYP-D3(BJ)/def2-TZVP.

For the monohydrate of 2-adamantanol, water may act as a proton donor or acceptor to the alcohol, forming in both cases a conventional O-H···O hydrogen bond. Because of the high-symmetry of 2-adamantanol, two equivalent isomers are obtained for each of the *gauche* and *anti* water-donor isomers (denoted *gauche*-Wd and *anti*-Wd). For the water acceptor, only the *gauche* isomer is doubly degenerate (*gauche*-Wa) but not the *C_s_*-symmetric *anti* form (*anti*-Wa). The optimized structures are shown in Figure 2, with the computational predictions collected in Table 2 (B2PLYP) and Table S3 (SM, B3LYP). 

The computational predictions were compared with the microwave spectrum obtained following the introduction of water in the gas line. This spectrum contains additional *μ*_a_ (R-branch) and *μ*_b_ (R and Q branches) rotational transitions with angular momentum quantum numbers *J* = 2–9 (*K*_−1_ < 5), corresponding to a single asymmetric rotor. Again, no torsional tunnelling effects were observed, and the spectrum was analysed with a semirigid-rotor Hamiltonian. The fitted rotational parameters and the measured transitions are shown in Table 2 and Table S4 (SI), respectively. 

The experiment undoubtedly established that water acts as a proton donor to 2-adamantanol in the monohydrate. However, the conformational assignment of the 2-adamantanol moiety is more difficult. The comparison of the rotational constants offers a slightly better agreement for the *anti*-Wd isomer for both the B3LYP and B2PLYP methods (i.e., relative differences of 0.3–1.2% for *gauche*-Wd and 0.1–0.9% for *anti*-Wd with B3LYP) but the differences are not conclusive. On the other hand, the harmonic prediction of the centrifugal distortion constants seems to suggest a better agreement for the *gauche*-Wd form. Since the *μ*_a_ and *μ*_b_ electric dipole moment components are relatively close in both isomers, the structural assignment of the 2-adamantanol monohydrate is presently not conclusive.

### 3.3. 2-Adamantanol Dimer

**Figure 3:**
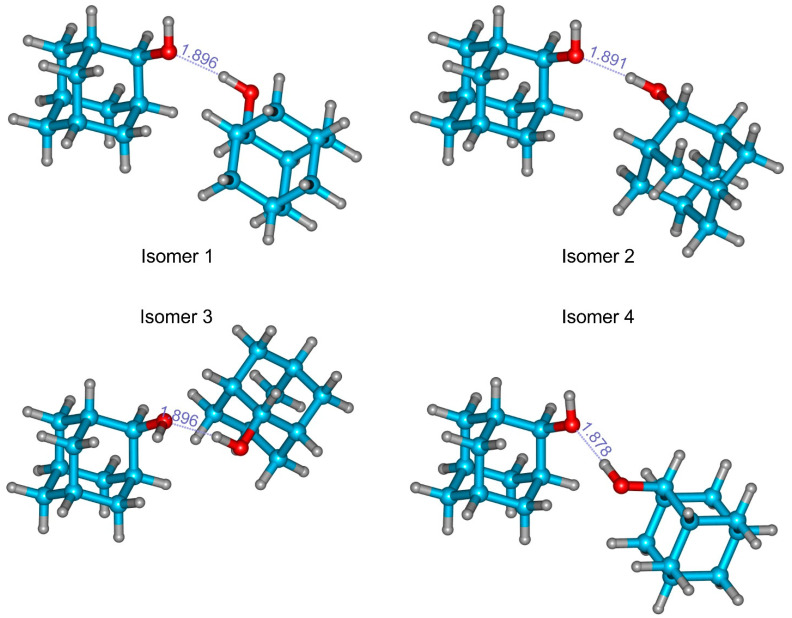
The lowest-lying isomers of the 2-adamantanol dimer are mostly comprised of *gauche*-*gauche* isomers. The O-H···O hydrogen bond is represented by a dotted line, together with the bonding distances predicted with B3LYP-D3(BJ)/def2-TZVP.

The investigation of the PES of the 2-adamantanol dimer initially used the B3LYP and B2PLYP methods with D3(BJ) empirical dispersion corrections. However, because of the larger computational cost of the B2LYP optimization for the dimer, the last method was restricted here to single-point energy calculations and the evaluation of the BSSE-corrected complexation energy, assuming the B3LYP geometries. Later, the most stable B3LYP structures were also reoptimized with the CAM-B3LYP-D3(BJ) and ωB97XD methods. As expected, the PES of the 2-adamantanol dimer is quite flat. The dimer is built on the moderately strong O-H···O hydrogen bond, but the internal rotation of any of the two aliphatic sidechains produces a large number of conformations quite similar in energy. Following the initial screening with MMFFs, a total of 58 plausible dimer geometries were found. The first 30 structures were then fully optimized with B3LYP. The six most stable isomers, falling within an energy window of only 1 kJ mol^−1^ (B3LYP), are presented in Table 3. Isomers 1 to 4 are shown in Figure 3 for illustration purposes. The two most stable structures display a *gauche*-*gauche* conformation (see 3D rotatable Figures S1 and S2 in SM), consistent with the structural preferences of the monomer. However, in some cases a *trans* conformation is predicted for one of the monomers, as in isomer 3. The structural data initially suggested that the aliphatic sidechains do not interact significatively in the dimer, as the closest contacts between aliphatic groups are larger than the van der Waals radii (i.e., *r*(H···H) > 2.30–2.35 Å).

**Figure 4:**
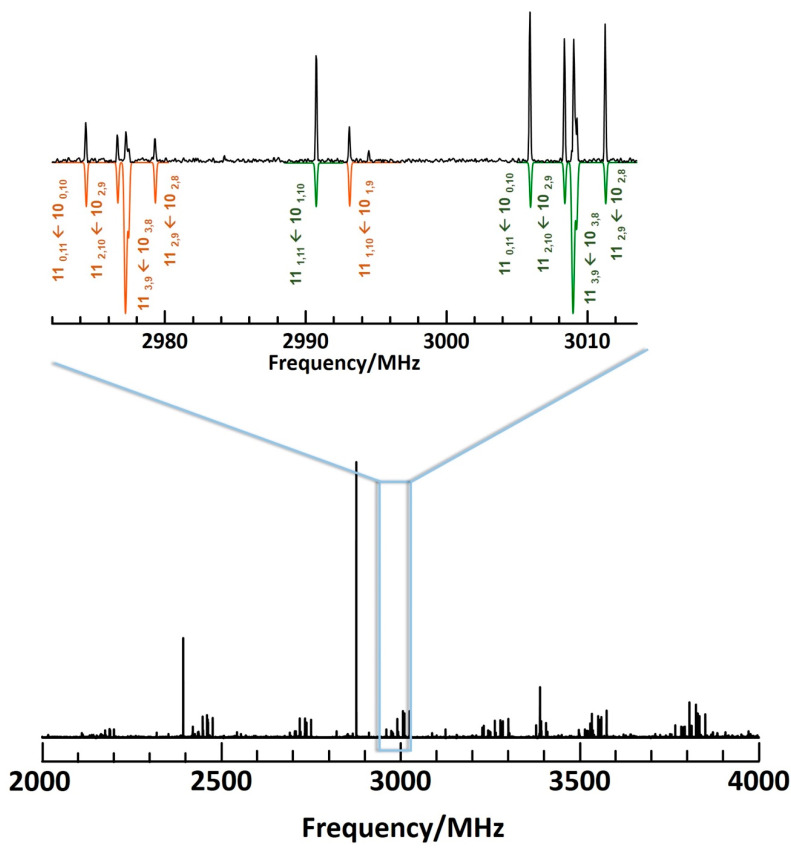
A view of the microwave spectrum of 2-adamantanol in the region 2–4 GHz (lower trace) and a 40 MHz expansion (upper trace) illustrating several typical rotational transitions corresponding to the two isomers of the 2-adamantanol dimer (isomer 1 in green; isomer 2 in orange).

The microwave spectrum provided precise empirical evidence on the matrix-free dimerization of 2-adamantanol. Noticeably, all predicted geometries converged into two observable dimers in the jet-cooled expansion, denoted isomers A and B in Table 3. Figure 4 shows a general view of the spectrum (2–4 GHz), together with an expanded section with several *J =* 11←*J* = 10 (*K*_−1_ = 0, 1, 2, 3) transitions. As observed in Figure 4, two sets of lines are clearly distinguished. Both spectra are mostly composed of R-branch μ_a_ transitions, but a few additional ^c^R lines can also be detected. The final experimental dataset comprised 88–124 transitions for each isomer, spanning angular momentum quantum numbers of *J* = 3–22 (*K*_−1_ < 6). Again, no tunnelling effects were detectable, so the dimer was fitted to a semirigid-rotor Hamiltonian. Actually, all observed transitions were reproduced to experimental accuracy with minor centrifugal distortion contributions, as only one or two distortion parameters turned determinable. The derived rotational parameters for the two dimers are shown in Table 3 (the weaker *μ*_c_ transitions were excluded from the final fit). The frequency measurements are presented in Tables S5 and S6 (SM), respectively.

The carriers of the spectrum were obtained by comparison of the experimental rotational constants with the computational predictions. However, the 2-adamantanol dimer offers a particularly difficult situation. The two isomers present quite similar values of the rotational constants, differing in less than 3 MHz (<1%) for all three inertial axes. These differences are of similar order compared with the computational uncertainties observed for the monomer and the monohydrate (<0.9%). Moreover, the electric dipole moment components and the harmonic centrifugal distortion constants are quite close for the most stable isomers and do not offer additional arguments for isomer discrimination. Despite this fact, a very good agreement is found in Table 3, suggesting that the observed species correspond to the two most stable isomers 1 and 2, predicted as being nearly isoenergetic (<1 kJ mol^−1^). The reoptimized CAM-B3LYP-D3(BJ) and wB97XD structures, shown in Table S7 (SM), offered a worse match with the experiment.

The jet populations of the two isomers of the 2-adamantanol dimer were estimated from relative intensity measurements. This calculation assumed a uniform instrumental response and a low-power linear fast-passage excitation regime [35,36], resulting in a quadratic dependence of the line intensities with the electric dipole moment components. Under these conditions, the predicted dipole moments of Table 3 (B3LYP-D3(BJ): *μ*_a_ = 3.12 D and 2.66 D, respectively) were used to estimate the relative isomer populations from a set of 10 ^a^ R transitions with *J* = 8–10 (Table S8, SM). The intensity measurements resulted in a population ratio of isomer 1–isomer 2 = 2.3(5). We did not attempt to calculate the relative energies of the two isomers because the populations may be affected by collisional conformational relaxation [51,52] and do not reflect the thermodynamic equilibrium [53]. Moreover, the effective conformational temperature is unknown. However, some information can be derived from previous jet experiments. In the case of hexanal [54], where twelve different conformations could be observed, the relative populations were reproduced for a conformational temperature of 135(11) K. Similarly, a model assuming full low-barrier conformational relaxation and a conformational temperature of 150 K reproduced the intensity observations in perillyl alcohol [55]. In the 2-adamantanol dimer, a conformational temperature of 135 K translates into an energy difference of 1.0 kJ mol^−1^, which is compatible with the B3LYP and B2PLYP calculations of Table 3 and Table S7 (SM). 

Some interconversion barriers between the predicted isomers have been calculated by global reaction route mapping [46] and intrinsic reaction coordinate calculations at the B3LYP-D3(BJ)/6-31g(d) level of calculation, followed by B3LYP-D3(BJ)/def2-TZVP reoptimization of the minima and transition states (atomic coordinates of isomers 1 and 2 in Tables S9 and S10, SM). The IRC path and interconversion barrier between isomers 1 and 2 shown in Figure S3 (SM) presents one transition state, with direct and reverse barriers of 2.4 and 3.6 kJ mol^−1^, respectively (B3LYP-D3(BJ)/def2-TZVP). Other conversion paths are characterized by two intermediate states, such as those between isomers 1 and 4 in Figure S4 (SM), with larger barrier heights of 4.7–5.8 kJ mol^−1^ (B3LYP-D3(BJ)/def2-TZVP). These barriers can be compared to previous empirical information on jet experiments. In molecules with a single torsional coordinate, the conformational relaxation threshold was estimated as ca. 4.8 kJ mol^−1^ [56], slightly larger than the predicted 1←2 conversion. For systems with multiple degrees of freedom, the potential energy surface, interconversion paths, barrier heights and cooling dynamics all affect jet populations, but the threshold barriers for effective relaxation are expected to be larger (<12 kJ mol^−1^) [51,52]. In this situation the predictions for the interconversion barriers are low but not conclusive, and we can only interpret empirically the observed jet populations as a result of the simultaneous formation of two nearly isoenergetic dimers and the absence of effective interconversion paths under our experimental conditions. Further details would require a comprehensive description of the full PES, which is out of the scope of this work.

### 3.4. Non-Covalent Interactions

The investigation of NCI in the monohydrate and the dimer used structural and electronic information. The O-H···O hydrogen bond in the water-donor monohydrate is regular, i.e., characteristically short and quite linear (B3LYP: *r_e_*(O···H) = 1.88–1.89 Å, ∠(O-H···O) = 168.3°–174.3°). These features are consistent with the cyclohexanol-H_2_O dimer (MP2: *r_e_*(O···H) = 1.88 Å, *r*_0_(O···H) = 1.928(5) Å) [20], the water dimer (VRT: *r_e_*(O···O) = 2.95 Å, *r*_0_(O···O)= 2.976 Å) [57], other acyclic aliphatic monohydrates [12,13,14,15,16] and high-order water clusters [34,58,59]. In all cases, the observations fall within the large margin of the O-H···O interaction distances observed in crystals (1.40–2.18 Å) [3,4]. 

The complexation energies for the monohydrate and the dimer of 2-adamantanol (B3LYP/B2PLYP, plus BSSE corrections) are collected in Table 2, Table 3 and Table S3 (SM). In the monohydrate, the complexation energy (B3LYP: −29.3 kJ mol^−1^; B2PLYP: −27.1 kJ mol^−1^) is comparable to the cyclohexanol–water adduct (B3LYP: −31.0 kJ mol^−1^; B2PLYP: −28.5 kJ mol^−1^; MP2: −22.9 kJ mol^−1^) [20]. On the other hand, the complexation energies are smaller than for aromatic monohydrates with several binding sites such as the thenyl (B3LYP: −33.2 kJ mol^−1^) [27] or furfuryl alcohols (B3LYP: −35.0 kJ mol^−1^) [25]. For the 2-adamantanol dimer, the complexation energies (B3LYP: −35.8/−35.6 kJ mol^−1^; B2PLYP: −32.7/−32.5 kJ mol^−1^) are slightly larger than in the cyclohexanol dimer (B3LYP: −33.9/−30.5 kJ mol^−1^) [21] but smaller than in the aromatic dimers of benzyl (B3LYP: −42.1 kJ mol^−1^) [23], thenyl (B3LYP: −46.9 kJ mol^−1^; B2PLYP: −41.8 kJ mol^−1^) [26] and furfuryl alcohol (B3LYP: −42.7 kJ mol^−1^; B2PLYP: −38.9 kJ mol^−1^) [26].

Additionally, an energy decomposition [7] was carried out using symmetry-adapted perturbation theory at the 2 + (3) level (optimized geometries of B3LYP-D3(BJ)/def2-TZVP), with results collected in Table 4. As expected, the largest attractive contributions to the 2-adamantanol monohydrate and dimer are electrostatic (58% and 47%, respectively). Interestingly, the monohydrate has a larger dispersive component (27%) than the dimer (19%), but this is considerably smaller than in aromatic dimers such as the benzyl mercaptan (54%) or thiophenol dimers (60%), where dispersion is larger than the electrostatic contribution. Table 4 includes also a comparison with the dispersive pyridine–methane dimer and the water and hydrogen sulfide dimers. A comparison with other thiol clusters and monohydrates is presented elsewhere [25,27]. 

**Figure 5:**
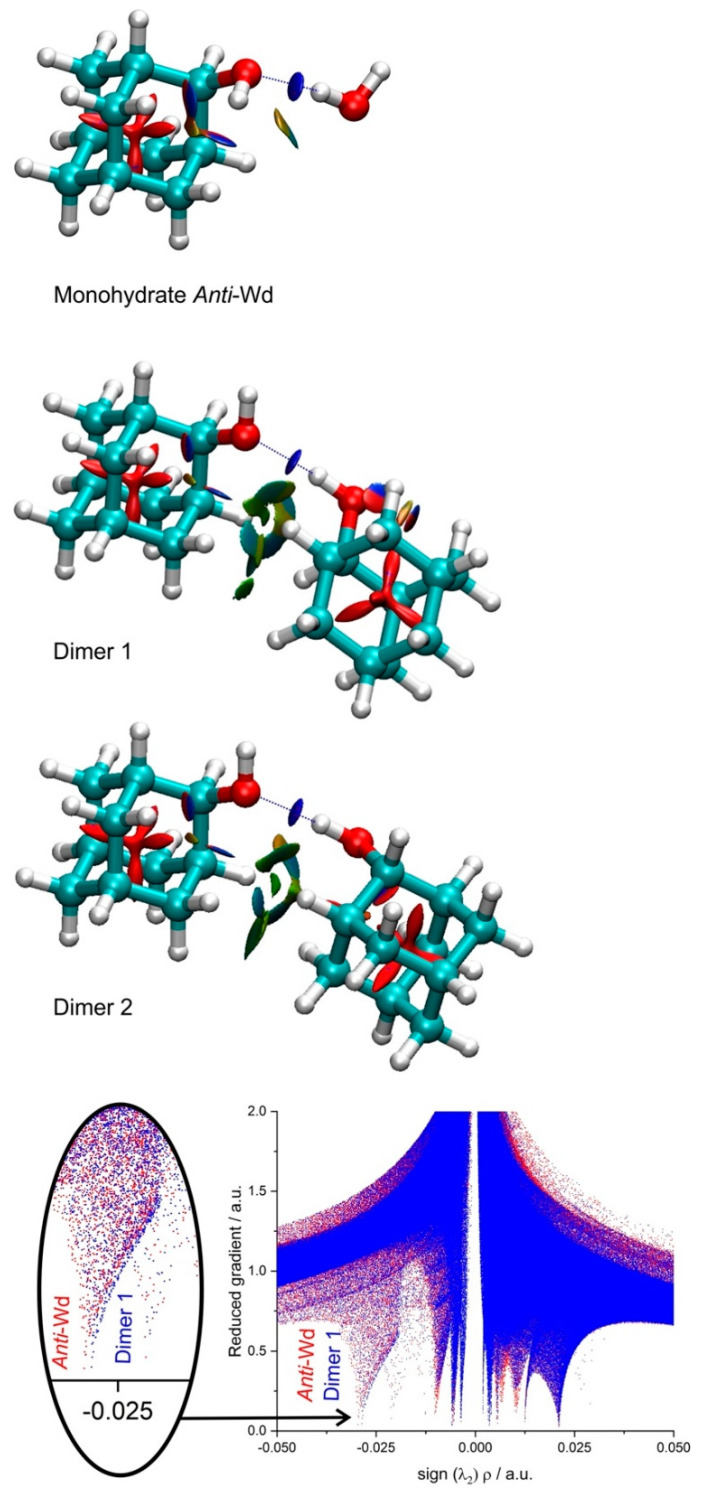
NCI plots [49] (upper panels) and reduced electronic density gradient (lowest panel) for the monohydrate and the two dimers of 2-adamantanol (isosurfaces with *s* = 0.5 a.u.). The NCI plot identifies attractive interactions (such as the O-H···O hydrogen bond) as blue shades, while green and red colors indicate, respectively, weak attractive interactions or repulsive interactions (such as ring critical points). The reduced electronic density of the lowest panel compares the attractive (negative minima) and repulsive (positive minima) interactions in the monohydrate (red dots) and isomer 1 (blue dots) of the 2-adamantanol dimer, dominated by the O-H···O interaction.

Non-covalent interactions were also analysed using the Johnson–Contreras NCIPlot topological analysis of the electron density (ρ(r)) [49], based on the reduced electronic density gradient $s\;\left(=\frac{1}{2{\left(3\pi^{2}\right)}^{\frac{1}{3}}}\frac{\left|\nabla \rho \right|}{\rho^{\frac{4}{3}}}\right)$. Figure 5 contains a representation of the reduced gradient (*s*) versus the signed electronic density $\left(\mathrm{sign}\;\left(\lambda_{2}\right)\;\rho \right)$ using the second eigenvalue $\left(\lambda_{2}\right)$ of the electron density Hessian, together with a spatial mapping of the non-covalent interactions in the title compounds (using a blue–green–red color code). Figure 5 identifies the primary O-H···O hydrogen bond interactions in the alcohols (blue surfaces along the O-H···O directions), simultaneously suggesting additional regions with minor interactions (green shades). As an example, intramolecular interactions caused by the lone pairs of the oxygen atom are apparent in the Figure, both in the dimer and the monohydrate. At the same time, intermolecular interactions are suggested for the monohydrate similarly involving the water oxygen lone pair and the C-H groups of 2-adamantanol. In the 2-adamantanol dimer, weak intermolecular interactions between the two aliphatic side chains are revealed, qualitatively attributing some attractive role to both sidechains on dimerization. Intramolecular repulsive regions (mostly inside the rings) are marked by red shades. The representation of the signed reduced electronic density gradient also compares the strength of the NCI in the monohydrate and the dimer, observing a close correspondence between the main O-H···O interactions in both dimers (most negative minima in the *s* vs ρ Figure). The reduced electronic density calculations thus complement the structural and energetic description of the 2-adamantanol dimers in Table 2, Table 3 and Table 4.

## 4. Discussion

The monohydrate and dimer of 2-adamantanol constitute model clusters combining a relatively strong electrostatically-dominated O-H···O hydrogen bond with a moderately-sized (10-carbon) highly-symmetric aliphatic sidechain. These dimers thus permit investigating several molecular aspects concerning non-covalent interactions, including the competition between the main hydroxyl hydrogen bond and the interactions associated to the aliphatic group, the balance of intra and intermolecular forces, the conformational equilibria in the jet, the presence of internal large-amplitude motions and the performance of DFT calculations. At the same time, the work represents an extension of the previous rotational investigations on the related monohydrate [20] and dimer [21] of cyclohexanol, offering grounds for a comparison with analog alcohol molecular systems. The monohydrates of cyclohexanol and 2-adamantanol similarly produce a single isomer, with the water molecule acting as a proton donor. This fact is readily explained by the formation of the dominant O-H···O interaction. However, the internal dynamics of both dimers is different. In the *gauche* cyclohexanol monohydrate the water molecule is involved in a concerted internal motion symmetrically inverting the hydroxyl and water orientations, as revealed in the rotational spectrum by the presence of tunneling doublings associated to a double-minimum potential barrier of 494 cm^−1^ (31% larger than in the monomer). In the 2-adamantanol monohydrate, the observed conformation is not conclusively determined, but the good agreement with the *anti* isomer is consistent with the absence of tunnelling effects in the spectrum. In the hypothesis that the monohydrate was adopting a *gauche* conformation, the absence of tunnelling effects might be explained as the result of a larger barrier or a larger reduced mass for the inversion vibration. The complexation energy in the 2-adamantanol monohydrate (B3LYP: −29.3 kJ mol^−1^; B2PLYP: −27.1 kJ mol^−1^) is estimated to be ca. 5–6% smaller than in cyclohexanol–water, which could be qualitatively related to the larger positive inductive effect of the side chain. However, the structural data show similar hydrogen bond geometries in both cases, suggesting that the strength of the hydrogen bond is not significantly altered.

The rotational observation of the dimer offers structural evidence with far more detail than the recognition of the O-H···O interaction noticed before [30]. The PES of the dimers is characteristically flat and difficult to assess computationally, both for cyclohexanol and 2-adamantanol. Interestingly, there is a reduction in complexity in the 2-adamantanol dimer PES compared to the cyclohexanol dimer, as only two isomers were observed for 2-adamantanol. This fact might also be associated to the axial/equatorial conformational multiplicity of cyclohexanol. The observed spectral intensities could be translated into jet populations, but the collisional mechanisms, clustering dynamics and dimer formation are still under-investigated and would require a comprehensive investigation of the PES and interconversion barriers, which are out of the scope of this work. The 2-adamantanol dimer offers 5-14% larger complexation energies than the cyclohexanol dimer, but, again, the structural data do not reflect important changes in the hydrogen bond between the two systems. The SAPT energy decomposition confirmed the expected electrostatic preference of the NCI in the 2-adamantanol dimers, as in related aliphatic alcohols. Finally, the analysis of the reduced electron density gradient suggests minor contributions from weak inter- and intramolecular interactions, more relevant for the formation of the homodimer.

While there is a good agreement between the experiment and the D3-corrected B3LYP and B2PLYP DFT models, we recognize some computational difficulties previously noticed in the investigation of the cyclohexanol dimer [21] and other weakly-bound clusters, i.e., the difficulty to locate all stable minima in automated conformational searches and the need for dispersion corrections, ultra-fine grids and tight convergence criteria for the conformational assignment of the observed. We should note also the worse performance of the CAM-B3LYP-D3BJ and ωB97XD methods in terms of structural properties. This fact requires further computational investigation but might indicate a favorable error compensation for the B3LYP-D3(BJ)/def2-TZVP calculation level. Concerning the quality of the energetic calculations, more detailed CCSD benchmarking calculations are required.

## 5. Conclusions

The emergence of broadband (chirped-pulse) fast-passage microwave techniques has boosted the scope and capacity of rotational investigations, offering insight into large, more complex chemical systems and NCI interactions. However, further evolution is associated with progress in the computational methods, required for the interpretation of complex spectra approaching the congestion limit. In this process, the combination of rotational and computational data offers empirical information effectively complementing vibrational and electronic spectroscopies. 

## Figures and Tables

**Table 1:** Rotational parameters for the 2-adamantanol monomer.

	Experiment	Theory B2PLYP-D3(BJ)	
	G*auche*	*Gauche*	*Anti*
*A*/MHz ^a^	1680.6888(30) ^d^	1691.9	1689.5
*B*/MHz	1197.8532(20)	1204.5	1197.5
*C*/MHz	1195.3429(18)	1202.3	1192.6
*κ*	−0.990(4)	−0.991	−0.980
*D_J_*/kHz	[ 0.] ^e^	0.0322	0.0310
*D_JK_*/kHz	0.280(89)	0.0303	0.0299
*D_K_*/kHz	−0.204(61)	−0.0094	−0.0080
*d*_1_/kHz	[ 0.]	0.0005	0.0010
*d*_2_/kHz	[ 0.]	0.0000	0.0006
|*μ*_a_|/D		0.48	1.75
|*μ*_b_|/D		0.93	0.72
|*μ*_c_|/D		0.96	0.00
Δ*E_ZPE_*/kJ mol^−1 b^		0.00	2.75
Δ*G*/kJ mol^−1^		0.00	2.47
*N* ^c^	25		
*σ*/kHz	7.5		

^a^ Rotational constants (*A*, *B*, *C*), Ray’s asymmetry parameter (*κ* = (2*B*–*A*–*C*)/(*A*–*C*)), Watson’s S-reduction centrifugal distortion constants (*D_J_*, *D_JK_*, *D_K_*, *d*_1_, *d*_2_) and electric dipole moments (*μ_α_*, *α* = a, b, c). ^b^ Relative electronic energy (Δ*E_ZPE_*) with zero-point correction and Gibbs energy (Δ*G*, 298 K, 1 atm). ^c^ Number of transitions (*N*) and rms deviation (*σ*) of the fit. ^d^ Standard errors in parentheses in units of the last digit. ^e^ Parameters in square brackets were fixed to zero.

**Table 2:** Rotational parameters for the 2-adamantanol monohydrate.

	Experiment	Theory B2PLYP-D3(BJ)			
		*Gauche*-Wd	*Anti*-Wd	*Gauche*-Wa	*Anti*-Wa
*A/*MHz ^a^	1511.8092(12) ^d^	1534.02	1524.14	1561.90	1481.44
*B/*MHz	690.17508(75)	684.93	694.66	654.28	738.62
*C/*MHz	662.22912(72)	659.66	666.48	635.52	696.20
*κ*	−0.934(1)	−0.942	−0.934	−0.959	−0.892
*D_J_*/kHz	0.3846(79)	0.2399	−0.6722	0.1968	0.1643
*D_JK_*/kHz	1.732(24)	7.2570	0.1143	0.3920	2.9012
*D_K_*/kHz	−1.721(36)	−7.2804	−0.7008	−0.3013	−2.8952
*d*_1_/kHz	−0.0366(65)	−0.0291	−3.4672	−0.0221	−0.0178
*d*_2_/kHz	0.0067(13)	0.0316	−0.6228	0.0018	0.0177
|*μ*_a_|/D		2.68	2.13	2.97	1.59
|*μ*_b_|/D		1.21	1.20	1.24	0.84
|*μ*_c_|/D		0.45	0.37	0.32	0.00
Δ*E_ZPE_*/kJ mol^−1 b^		0.00	1.98	3.25	6.34
Δ*G*/kJ mol^−1^		0.00	4.24	2.50	11.12
Δ*E*_c_/kJ mol^−1^		−25.31	−27.11	−22.34	−21.51
*N* ^c^	60				
*σ*/kHz	10.6				

^a^ Rotational constants (*A*, *B*, *C*), Ray’s asymmetry parameter (*κ* = (2*B*-*A*–*C*)/(*A*–*C*)), Watson’s S-reduction centrifugal distortion constants (*D_J_*, *D_JK_*, *D_K_*, *d*_1_, *d*_2_) and electric dipole moments (*μ_α_*, *α* = a, b, c). ^b^ Relative electronic energies (Δ*E_ZPE_*) with zero-point correction, Gibbs energy (Δ*G*, 298K, 1 atm) and complexation energy (Δ*E*_c_). ^c^ Number of transitions (*N*) and rms deviation (*σ*) of the fit. ^d^ Standard errors in parentheses in units of the last digit.

**Table 3:** Rotational parameters for the 2-adamantanol dimer.

	Experiment		Theory					
	Isomer A	Isomer B	Isomer 1-CS1	Isomer 2-CS4	Isomer 3	Isomer 4	Isomer 5	Isomer 6
*A/*MHz ^a^	701.117(17) ^e^	702.79(27)	703.95	702.74	701.46	709.22	701.84	712.05
*B/*MHz	138.34738(18)	136.84142(21)	140.03	139.42	141.81	139.83	145.30	138.32
*C/*MHz	135.18537(18)	133.80126(23)	136.84	136.13	138.49	137.08	142.18	135.81
*κ*	−0.9888(4)	−0.9893(8)	−0.989	−0.988	−0.988	−0.990	−0.989	−0.991
*D_J_/*kHz	0.00643(18)	0.00471(23)	0.0063	0.0045	0.0061	0.0046	0.0057	0.0065
*D_JK_/*kHz	−0.0169(54)		−0.0150	0.0142	−0.0067	0.0037	−0.0121	−0.0222
*D_K_/*kHz			0.0532	0.0104	0.0361	0.0203	0.0426	0.0808
*d*_1_*/*kHz			−0.0003	−0.0002	−0.0003	−0.0002	−0.0003	−0.0004
*d*_2_*/*kHz			0.0000	0.0000	0.0000	0.0000	0.0000	0.0000
|*μ*_a_|/D			3.12	2.66	2.61	2.60	2.33	2.86
|*μ*_b_|/D			0.23	0.31	0.73	0.24	1.11	0.46
|*μ*_c_|/D			1.10	1.21	0.79	1.84	2.07	1.83
*N* ^b^	124	88						
*σ/*kHz	13.2	10.5						
B3LYP-D3 ^c^								
Δ*E_ZPE_/*kJ mol^−1^			0.00	0.00	1.00	0.69	1.09	0.83
Δ*G*_298 K_*/*kJ mol^−1^			0.97	0.00	2.03	1.68	3.18	0.69
Δ*E_c_/*kJ mol^−1^			−35.77	−35.61	−36.86	−35.19	−34.64	−34.48
B2PLYP-D3 ^d^								
Δ*E_ZPE_/*kJ mol^−1^			0.00	0.04	3.05	0.43	2.32	0.32
Δ*E*_c_*/*kJ mol^−1^			−32.72	−32.47	−33.76	−32.17	−31.09	−31.46

^a^ Rotational constants (*A*, *B*, *C*), Ray’s asymmetry parameter (*κ* = (2*B*–*A*–*C*)/(*A*–*C*)), Watson’s S-reduction centrifugal distortion constants (*D_J_*, *D_JK_*, *D_K_*, *d*_1_, *d*_2_) and electric dipole moments (*μ_α_*, *α* = a, b, c). ^b^ Number of transitions (*N*) and rms deviation (*σ*) of the fit. ^c^ B3LYP-D3(BJ) energetics: relative electronic energies (Δ*E_ZPE_*) with zero-point correction, Gibbs energy (Δ*G*, 298 K, 1 atm) and complexation energy (Δ*E*_c_). ^d^ B2PLYP-D3(BJ) energetics: relative electronic energies (Δ*E_ZPE_*) with B3LYP-D3(BJ) zero-point corrections and complexation energy (Δ*E*_c_). ^e^ Standard errors in parentheses in units of the last digit.

**Table 4:** Binding energy decomposition for the dimer and monohydrate of 2-adamantanol, and comparison with several alcohol and thiol dimers and the van der Waals dimer pyridine–methane. Clusters are ordered by interaction energy, which is decomposed into electrostatic (Δ*E*_elec_), inductive (multipole interactions/charge transfer, Δ*E*_ind_), exchange repulsion (Δ*E*_exch_) and dispersion (Δ*E_disp_*) energy terms. The calculation used SAPT(2) + 3/aug-cc-pVDZ//B3LYP-D3(BJ)/def2-TZVP (all values in kJ mol^−1^).

Cluster	Δ*E*_elec_	Δ*E*_disp_	Δ*E*_ind_	Δ*E*_exch_	Δ*E*_total_	Δ*E*_S22_
(Benzyl alcohol)_2_^a^	−58.7 [44.5%] ^k^	−54.6 [41.4%]	−18.6 [14.1%]	89.8	−42.1	
(Furfuryl alcohol)_2_ ^b^	−61.7 [49.7%]	−21.5 [17.3%]	−41.0 [33.0%]	85.7	−38.5	
(Benzyl mercaptan)_2_^c^	−39.3 [34.4%]	−61.1 [53.6%]	−13.6 [12.0%]	78.7	−35.3	
(2-Adamantanol)_2_ Isomer 1 ^d^	−44.8 [46.6% ]	−16.2 [19.2%]	−35.2 [51.7%]	63.3	−32.9	
(2-Adamantanol)_2_ Isomer 2 ^d^	−45.6 [46.9%]	−16.7 [19.8%]	−35.2 [51.9%]	64.9	−32.9	
(Cyclohexanol)_2_^e^	−46.5 [51.8%]	−16.6 [18.5%]	−26.6 [29.7%]	60.5	−29.2	
(Phenol)_2_^f^	−41.8 [48.3%]	−28.8 [18.4%]	−15.9 [33.3%]	58.9	−27.6	−29.5 ^l^
(Thiophenol)_2_ PD1-*trans* ^g^	−24.9 [31.0%]	−47.9 [59.5%]	−7.7 [9.5%]	54.6	−25.9	
2-Adamantanol ··· H_2_O ^d^	−42.3 [57.6%]	−14.6 [26.7%]	−16.5 [41.3%]	49.9	−23.5	
(H_2_O)_2_^h^	−35.7 [63.5%]	−9.5 [16.8%]	−11.1 [19.8%]	37.7	−18.6	−21.0 ^l^
(H_2_S)_2_^i^	−12.1 [49.0%]	−7.8 [31.7%]	−4.7 [19.3%]	19.2	−5.4	
Pyridine–methane ^j^	−3.0 [20.6%]	−10.9 [74.6%]	−0.7 [4.8%]	9.4	−5.2	

^a^ Ref. [23] ^b^ Ref. [26]. ^c^ Ref. [60]. ^d^ This work. ^e^ Ref. [21]. ^f^ Ref. [22]. ^g^ Ref. [61]. ^h^ Ref. [57]. ^i^ Ref. [62]. ^j^ Ref. [63]. ^k^ Relative percentage contribution to the attractive interactions (Δ*E*_elec_ + Δ*E*_disp_ + Δ*E*_ind_). ^l^ Ref. [64].

## Data Availability

The data presented in this study are available in the supplementary material.

## Supplementary Materials

The following are available online at https://www.mdpi.com/article/10.3390/molecules27082584/s1, Figure S1: 3D-rotatable image of isomer 1 of the 2-adamantanol dimer (B3LYP-D3(BJ)/def2-TZVP), Figure S2: 3D-rotatable image of isomer 2 of the 2-adamantanol dimer (B3LYP-D3(BJ)/def2-TZVP), Figure S3: Interconversion barrier between isomers 1 (left) and 2 (right) of the 2-adamantanol dimer, using GRRM/IRC at the B3LYP-D3/6-31g(d) level. Reoptimization of the minima and transition states with B3LYP-D3/def2-TZVP gives barriers of 2.4 and 3.6 kJ mol^−1^, Figure S4: Interconversion barrier between isomers 1 (left) and 4 (right) of the 2-adamantanol dimer, using GRRM/IRC at the B3LYP-D3/6-31g(d) level. Reoptimization of the minima and transition states with B3LYP-D3/def2-TZVP gives barriers of 3.1–4.4 kJ mol−1, Table S1: Rotational parameters of 2-adamantanol and comparison with the B3LYP-D3(BJ)/def2-TZVP predictions, Table S2: Rotational transitions of the observed gauche conformer of 2-adamantanol (Freq) and differences between observed and calculated transitions (o-c) for the fit of Table 1, Table S3: Rotational parameters of the 2-adamantanol-water dimer and comparison with the B3LYP-D3(BJ)/def2-TZVP predictions, Table S4: Rotational transitions of the 2-adamantanol-water monohydrate (Freq.) and differences between observed and calculated transitions (o-c) for the fit of Table 2, Table S5: Rotational transitions of isomer 1 of the 2-adamantanol dimer (Freq.) and differences between observed and calculated transitions (o-c) for the fit of Table 3, Table S6: Rotational transitions of isomer 2 of the 2-adamantanol dimer (Freq.) and differences between observed and calculated transitions (o-c) for the fit of Table 3, Table S7: Comparison of the structural and energetic results for the two lowest-lying isomers of the 2-adamantanol dimer using B3LYP-D3(BJ), CAM-B3LYP-D3(BJ) and wB97XD (a def2-TZVP basis set as used in all cases). The experimental results correspond to the fit of Table 3, Table S8: Relative intensity measurements for a set of aR rotational transitions of isomers 1 and 2 of the 2-adamantanol dimer. The population ratio was calculated with the B3LYP-D3(BJ) electric dipole moments of Table 3, Table S9: Atomic coordinates for isomer 1 of the 2-adamantanol dimer in the principal inertial axes system (B3LYP-D3(BJ)/def2-TZVP), Table S10: Atomic coordinates for isomer 2 of the 2-adamantanol dimer in the principal inertial axes system (B3LYP-D3(BJ)/def2-TZVP).
