# Supplementary material for: Hydrogen Bonding in the Dimer and Monohydrate of 2-Adamantanol: A Test Case for Dispersion-Corrected Density Functional Methods
Source: Molecules. 2022 Apr 17;27(8):2584. doi: 10.3390/molecules27082584 (PMC9030514; doi:10.3390/molecules27082584)
Supplement: Supplementary file 1 [file molecules-27-02584-s001.zip › molecules-1676368-supplementary.pdf]

Hydrogen Bonding in the Dimer and Monohydrate of 2-Adamantanol: A Test Case for Dispersion-Corrected Density Functionals

SUPPORTING INFORMATION

Marcos Juanes [1], Rizalina Tama Saragi [1], Cristóbal Pérez [1], Luca Evangelisti [2], Lourdes Enríquez [3], Martín Jaraíz [3] and Alberto Lesarri [1,*]

[1] Departamento de Química Física y Química Inorgánica, Facultad de Ciencias - I.U. CINQUIMA, Universidad de Valladolid, Paseo de Belén, 7, 47011 Valladolid (Spain)

[2] Dipartimento di Chimica ''G. Ciamician'', Università di Bologna, Via Selmi 2, 40126 Bologna (Italy)

[3] Departamento de Electrónica, Escuela de Ingeniería de Telecomunicaciones, Universidad de Valladolid, Paseo de Belén, 15, 47011 Valladolid (Spain)

* Correspondence: alberto.lesarri@uva.es

LIST OF SUPPORTING FIGURES AND TABLES

Figure S1. 3D-rotatable image of isomer 1 of the 2-adamantanol dimer (B3LYP-D3(BJ)/def2-TZVP).

Figure S2. 3D-rotatable image of isomer 2 of the 2-adamantanol dimer (B3LYP-D3(BJ)/def2-TZVP).

Figure S3. Interconversion barrier between isomers 1 (left) and 2 (right) of the 2-adamantanol dimer, using GRRM/IRC at the B3LYP-D3/6-31g(d) level. Reoptimization of the minima and transition states with B3LYP-D3/def2-TZVP gives barriers of 2.4 and 3.6 kJ mol^{-1}.

Figure S4. Interconversion barrier between isomers 1 (left) and 4 (right) of the 2-adamantanol dimer, using GRRM/IRC at the B3LYP-D3/6-31g(d) level. Reoptimization of the minima and transition states with B3LYP-D3/def2-TZVP gives barriers of 3.1-4.4 kJ mol^{-1}.

Table S1. Rotational parameters of 2-adamantanol and comparison with the B3LYP-D3(BJ)/def2-TZVP predictions.

Table S2. Rotational transitions of the observed *gauche* conformer of 2-adamantanol (*Freq*) and differences between observed and calculated transitions (*o-c*) for the fit of Table 1.

Table S3. Rotational parameters of the 2-adamantanol-water dimer and comparison with the B3LYP-D3(BJ)/def2-TZVP predictions.

Table S4. Rotational transitions of the 2-adamantanol-water monohydrate (*Freq*.) and differences between observed and calculated transitions (*o-c*) for the fit of Table 2.

Table S5. Rotational transitions of isomer 1 of the 2-adamantanol dimer (*Freq*.) and differences between observed and calculated transitions (*o-c*) for the fit of Table 3.

Table S6. Rotational transitions of isomer 2 of the 2-adamantanol dimer (*Freq*.) and differences between observed and calculated transitions (*o-c*) for the fit of Table 3.

Table S7. Comparison of the structural and energetic results for the two lowest-lying isomers of the 2-adamantanol dimer using B3LYP-D3(BJ), CAM-B3LYP-D3(BJ) and wB97XD (a def2-TZVP basis set as used in all cases). The experimental results correspond to the fit of Table 3.

Table S8. Relative intensity measurements for a set of ^{a}R rotational transitions of isomers 1 and 2 of the 2-adamantanol dimer. The population ratio was calculated with the B3LYP-D3(BJ) electric dipole moments of Table 3.

Table S9. Atomic coordinates for isomer 1 of the 2-adamantanol dimer in the principal inertial axes system (B3LYP-D3(BJ)/def2-TZVP).

Table S10. Atomic coordinates for isomer 2 of the 2-adamantanol dimer in the principal inertial axes system (B3LYP-D3(BJ)/def2-TZVP).

Figure S1. 3D-rotatable image of isomer 1 of the 2-adamantanol dimer (B3LYP-D3(BJ)/def2-TZVP).

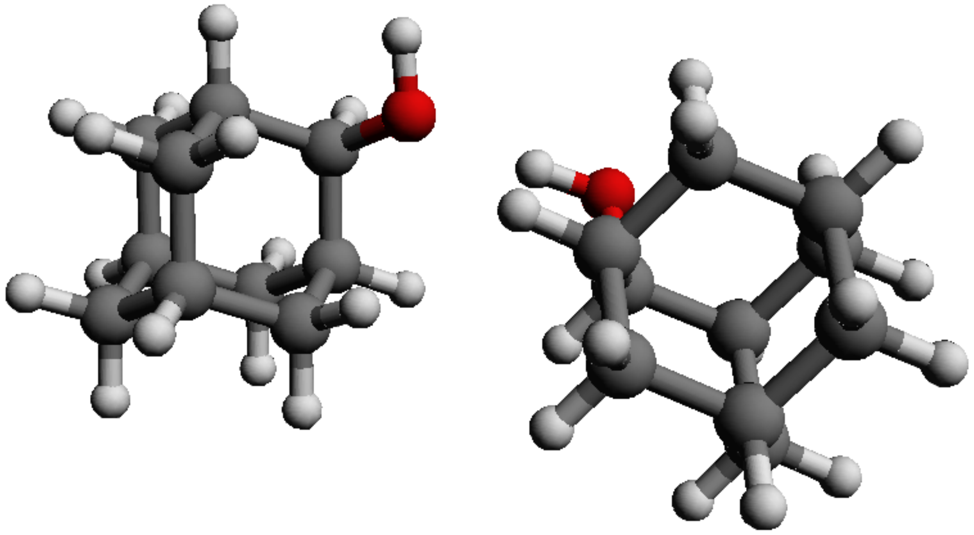

Figure S2. 3D-rotatable image of isomer 2 of the 2-adamantanol dimer (B3LYP-D3(BJ)/def2-TZVP).

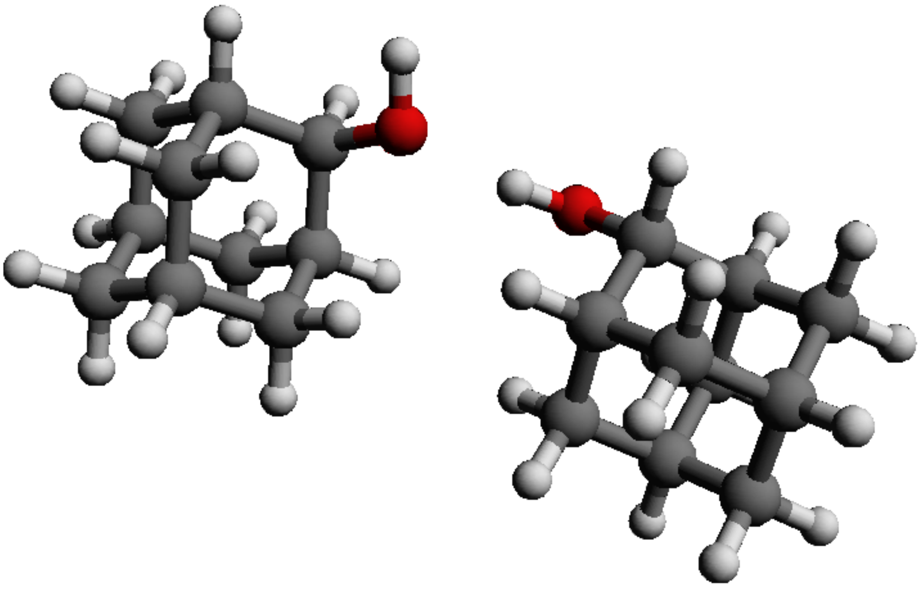

Figure S3. Interconversion barrier between isomers 1 (left) and 2 (right) of the 2-adamantanol dimer, using GRRM/IRC at the B3LYP-D3/6-31g(d) level. Reoptimization of the minima and transition states with B3LYP-D3/def2-TZVP gives barriers of 2.4 and 3.6 kJ mol^{-1}.

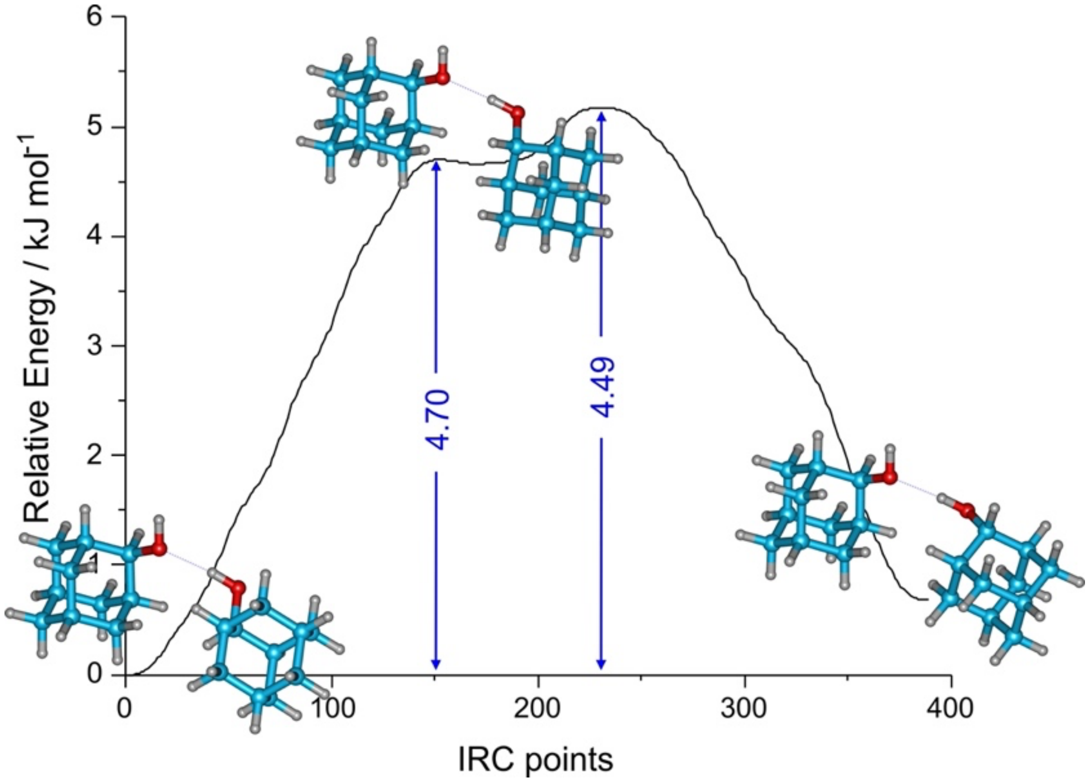

Figure S4. Interconversion barrier between isomers 1 (left) and 4 (right) of the 2-adamantanol dimer, using GRRM/IRC at the B3LYP-D3/6-31g(d) level. Reoptimization of the minima and transition states with B3LYP-D3/def2-TZVP gives barriers of 3.1-4.4 kJ mol^{-1}.

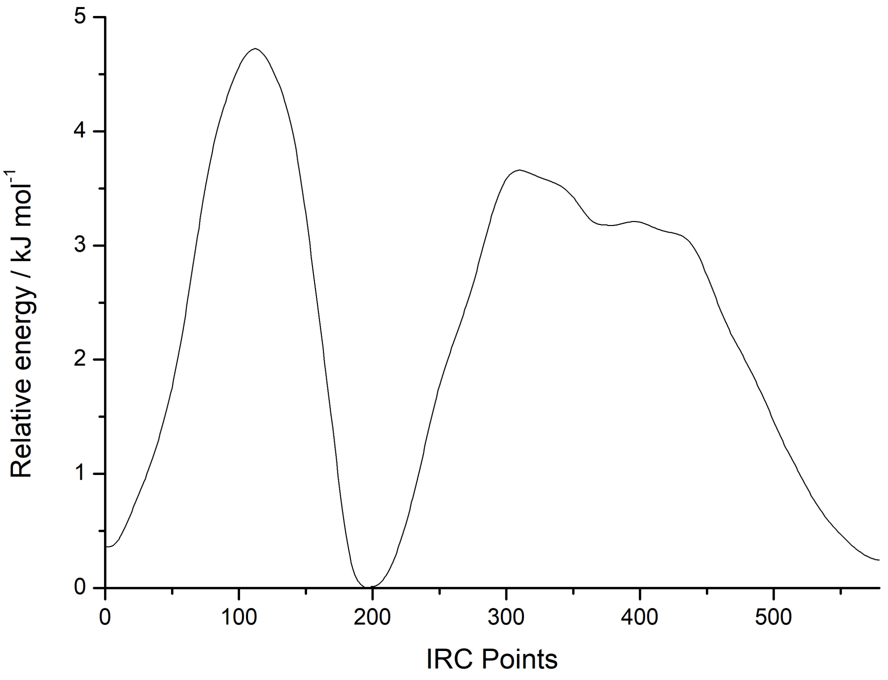

Table S1. Rotational parameters of 2-adamantanol and comparison with the B3LYP-D3(BJ)/def2-TZVP predictions.

	Experiment	B3LYP-D3(BJ)	
	Gauche	*Gauche*	*Anti*
A / MHz [a]	1680.6888(30) [d]	1688.38	1686.04
B / MHz	1197.8532(20)	1201.19	1194.68
C / MHz	1195.3429(18)	1199.03	1189.83
κ	-0.990(4)	-0.991	-0.980
D_J / kHz	[0.] [e]	0.0321	0.0312
D_{JK} / kHz	0.280(89)	0.0314	0.0274
D_K / kHz	-0.204(61)	-0.0105	-0.0056
d_1 / kHz	[0.]	0.0009	0.0008
d_2 / kHz	[0.]	0.0005	0.0006
$\|\mu_a\|$ / D		0.50	1.74
$\|\mu_b\|$ / D		0.90	0.72
$\|\mu_c\|$ / D		0.97	0.00
ΔE_{ZPE} / kJ mol^{-1} [b]		0.00	2.05
$\Delta G_{298\ K}$ / kJ mol^{-1}		0.00	1.80
N [c]	25		
σ / kHz	7.5		

[a]Rotational constants (A, B, C), Ray's asymmetry parameter ($\kappa = (2B-A-C)/(A-C)$), Watson's S-reduction centrifugal distortion constants (D_J, D_{JK}, D_K, d_1, d_2) and electric dipole moments (μ_α, α = a, b, c). [b]Relative electronic energy with zero-point correction (ΔE_{ZPE}) and Gibbs energy (ΔG, 298K, 1 atm). [c]Number of fitted transitions and rms deviation of the fit. [d]Standard errors in parentheses in units of the last digit. [e]Parameters in square brackets were fixed to zero.

Table S2. Rotational transitions of the observed *gauche* conformer of 2-adamantanol (*Freq*) and differences between observed and calculated transitions (*o-c*) for the fit of Table 1.

J'	K_{-1}'	K_{+1}'	J''	K_{-1}''	K_{+1}''	*Freq. / MHz*	*o-c / MHz*
1	0	1	0	0	0	2393.1875	-0.0086
7	3	4	7	2	5	2419.5000	-0.0048
6	3	3	6	2	4	2419.9250	0.0129
5	3	2	5	2	3	2420.1625	-0.0096
1	1	1	0	0	0	2876.0375	0.0060
4	4	0	4	3	1	3388.6125	-0.0010
2	0	2	1	1	1	4303.5500	0.0026
5	5	1	5	4	1	4356.7875	-0.0086
2	1	2	1	1	1	4783.8750	-0.0060
2	0	2	1	0	1	4786.3875	0.0048
2	1	1	1	1	0	4788.9125	0.0110
2	1	2	1	0	1	5266.7125	-0.0037
6	6	1	6	5	1	5324.9750	-0.0077
6	6	0	6	5	1	5324.9750	-0.0077
6	6	1	6	5	2	5324.9750	-0.0077
7	6	2	7	5	3	5324.9750	0.0426
3	1	3	2	2	0	5719.7625	-0.0075
3	1	2	2	2	1	5734.8250	-0.0162
2	2	1	1	1	0	6237.4000	-0.0063
2	2	0	1	1	1	6239.9250	-0.0013
3	0	3	2	1	2	6699.2250	0.0091
3	1	3	2	1	2	7175.8250	0.0095
3	0	3	2	0	2	7179.5500	0.0004
3	1	2	2	1	1	7183.3500	0.0038
3	1	3	2	0	2	7656.1500	0.0009

Table S3. Rotational parameters of the 2-adamantanol-water dimer and comparison with the B3LYP-D3(BJ)/def2-TZVP predictions.

	Experiment	B3LYP-D3(BJ)			
		Gauche-Wd	*Anti-Wd*	*Gauche-Wa*	*Anti-Wa*
A / MHz [a]	1511.8092(12) [d]	1530.18	1525.44	1556.88	1477.91
B / MHz	690.17508(75)	685.53	689.11	655.05	738.14
C / MHz	662.22912(72)	659.98	662.32	635.87	695.64
κ	-0.934(1)	-0.941	-0.938	-0.958	-0.891
D_J / kHz	0.3846(79)	0.1712	0.1477	0.2584	0.1303
D_{JK} / kHz	1.732(24)	2.0706	1.0529	0.2536	0.1365
D_K / kHz	-1.721(36)	-2.0364	-1.0312	-0.1456	-0.0965
d_1 / kHz	-0.0366(65)	-0.0185	-0.0160	-0.0301	-0.0175
d_2 / kHz	0.0067(13)	0.0107	0.0062	0.0021	0.0025
$\|\mu_a\|$ / D		2.78	2.27	2.77	1.58
$\|\mu_b\|$ / D		1.23	1.21	1.22	0.82
$\|\mu_c\|$ / D		0.45	0.34	0.49	0.00
ΔE_{ZPE} / kJ mol^{-1} [b]		0.00	1.46	4.12	6.65
$\Delta G_{298\,K}$ / kJ mol^{-1}		0.00	1.63	1.62	5.38
ΔE_C / kJ mol^{-1}		-27.66	-29.25	-23.47	-22.80
N [c]	60				
σ / kHz	10.6				

[a]Rotational constants (A, B, C), Ray's asymmetry parameter ($\kappa = (2B-A-C)/(A-C)$), Watson's S-reduction centrifugal distortion constants (D_J, D_{JK}, D_K, d_1, d_2) and electric dipole moments (μ_α, α = a, b, c). [b]Relative electronic energy with zero-point correction (ΔE_{ZPE}), Gibbs energy (ΔG, 298K, 1 atm) and complexation energy (ΔE_c). [c]Number of fitted transitions and rms deviation of the fit. [d]Standard errors in parentheses in units of the last digit.

Table S4. Rotational transitions of the 2-adamantanol-water monohydrate (*Freq.*) and differences between observed and calculated transitions (*o-c*) for the fit of Table 2.

J'	K_{-1}'	K_{+1}'	J''	K_{-1}''	K_{+1}''	*Freq. / MHz*	*o-c / MHz*
4	2	2	4	1	3	2378.8375	-0.0074
3	2	2	3	1	3	2591.0375	-0.0118
4	2	3	4	1	4	2647.7125	0.0000
2	1	2	1	1	1	2676.8500	0.0056
2	0	2	1	0	1	2704.1000	0.0047
5	2	4	5	1	5	2718.9125	0.0137
2	1	1	1	1	0	2732.7500	0.0160
6	2	5	6	1	6	2804.7750	-0.0137
3	0	3	2	1	2	3259.9875	-0.0018
2	1	2	1	0	1	3498.4750	-0.0019
3	1	3	2	1	2	4014.8125	-0.0005
3	0	3	2	0	2	4054.3750	0.0039
3	2	2	2	2	1	4057.1375	0.0086
3	2	1	2	2	0	4059.9375	0.0073
8	3	5	8	2	6	4080.0000	0.0041
3	1	2	2	1	1	4098.6375	0.0054
7	3	4	7	2	5	4117.2000	0.0029
6	3	3	6	2	4	4143.2125	0.0171
5	3	2	5	2	3	4160.0375	0.0095
4	3	1	4	2	2	4169.9250	0.0103
5	3	3	5	2	4	4184.0875	-0.0039
6	3	4	6	2	5	4190.7000	0.0052
7	3	5	7	2	6	4201.1625	-0.0090
8	3	6	8	2	7	4216.6625	0.0080
9	3	7	9	2	8	4238.3500	0.0112
5	1	4	4	2	3	4460.1375	0.0211
4	0	4	3	1	3	4647.7000	-0.0143
3	1	3	2	0	2	4809.1875	-0.0072
2	2	1	1	1	0	5197.6250	-0.0072
2	2	0	1	1	1	5226.2750	-0.0040
7	2	6	6	3	3	5269.1500	0.0127
6	1	6	5	2	3	5281.7000	0.0067
4	1	4	3	1	3	5352.2625	0.0094
7	2	5	6	3	4	5356.3250	-0.0097
4	0	4	3	0	3	5402.5375	-0.0005
4	2	3	3	2	2	5408.9250	0.0087
4	3	2	3	3	1	5410.7875	0.0250
4	3	1	3	3	0	5410.7875	-0.0410
4	2	2	3	2	1	5415.9125	0.0129
4	1	3	3	1	2	5463.9625	0.0103

6	1	5	5	2	4	5891.8875	0.0091
5	0	5	4	1	4	6043.4000	-0.0097
4	1	4	3	0	3	6107.0750	-0.0017
7	1	7	6	2	4	6506.7875	0.0002
3	2	2	2	1	1	6522.0250	-0.0021
3	2	1	2	1	2	6609.3750	0.0101
5	1	5	4	1	4	6689.0250	0.0118
5	0	5	4	0	4	6747.9500	0.0015
5	2	4	4	2	3	6760.2000	0.0006
5	4	1	4	4	0	6763.0875	-0.0219
5	3	3	4	3	2	6763.9625	-0.0048
5	3	2	4	3	1	6764.1875	-0.0106
5	2	3	4	2	2	6774.0875	0.0025
5	1	4	4	1	3	6828.4500	-0.0255
7	1	6	6	2	5	7335.1750	0.0097
5	1	5	4	0	4	7393.5500	-0.0018
6	0	6	5	1	5	7444.4250	-0.0202
7	5	3	7	4	4	7517.1375	-0.0221
6	5	2	6	4	3	7518.3875	0.0160
4	2	3	3	1	2	7832.3000	-0.0113

Table S5. Rotational transitions of isomer 1 of the 2-adamantanol dimer (*Freq.*) and differences between observed and calculated transitions (*o-c*) for the fit of Table 3.

J'	K_{-1}'	K_{+1}'	J''	K_{-1}''	K_{+1}''	*Freq. / MHz*	*o-c / MHz*
8	1	8	7	1	7	2175.3250	-0.0146
8	0	8	7	0	7	2187.1250	-0.0096
8	2	7	7	2	6	2188.0875	0.0000
8	3	5	7	3	4	2188.3875	-0.0276
8	2	6	7	2	5	2189.2000	-0.0016
8	1	7	7	1	6	2200.6375	0.0105
9	1	9	8	1	8	2447.1625	-0.0090
9	0	9	8	0	8	2460.1875	0.0015
9	2	8	8	2	7	2461.5375	-0.0005
9	3	7	8	3	6	2462.0000	0.0075
9	3	6	8	3	5	2462.0000	-0.0085
9	2	7	8	2	6	2463.1250	-0.0029
9	1	8	8	1	7	2475.6375	0.0239
10	1	10	9	1	9	2718.9625	-0.0117
10	0	10	9	0	9	2733.1250	0.0062
10	2	9	9	2	8	2734.9625	-0.0047
10	4	7	9	4	6	2735.4750	-0.0018
10	3	8	9	3	7	2735.6125	0.0224
10	3	7	9	3	6	2735.6125	-0.0051
10	2	8	9	2	7	2737.1500	-0.0001
10	1	9	9	1	8	2750.5750	0.0083
11	1	11	10	1	10	2990.7375	-0.0075
11	0	11	10	0	10	3005.9250	0.0039
11	2	10	10	2	9	3008.3625	-0.0103
11	4	8	10	4	7	3009.0375	-0.0151
11	4	7	10	4	6	3009.0375	-0.0154
11	3	9	10	3	8	3009.2750	0.0751
11	3	8	10	3	7	3009.2750	0.0302
11	2	9	10	2	8	3011.2750	-0.0025
11	1	10	10	1	9	3025.4875	0.0053
12	1	12	11	1	11	3262.4625	-0.0187
12	0	12	11	0	11	3278.5875	0.0056
12	2	11	11	2	10	3281.7625	0.0100
12	4	8	11	4	7	3282.6250	-0.0119
12	3	10	11	3	9	3282.8375	0.0149
12	3	9	11	3	8	3282.8375	-0.0548
12	2	10	11	2	9	3285.5125	-0.0060
12	1	11	11	1	10	3300.3481	-0.0074
13	1	13	12	1	12	3534.1875	0.0068
13	0	13	12	0	12	3551.1000	0.0089

13	2	12	12	2	11	3555.1000	-0.0036
13	6	8	12	6	6	3556.1000	0.0471
13	4	10	12	4	9	3556.2500	0.0209
13	4	9	12	4	8	3556.2500	0.0201
13	3	11	12	3	10	3556.4500	-0.0084
13	3	10	12	3	9	3556.5750	0.0119
13	2	11	12	2	10	3559.8750	-0.0059
13	1	12	12	1	11	3575.1750	-0.0071
14	1	14	13	1	13	3805.8375	-0.0035
14	0	14	13	0	13	3823.4250	-0.0146
14	2	13	13	2	12	3828.4250	0.0008
14	5	9	13	5	8	3829.6625	-0.0214
14	4	10	13	4	9	3829.8250	-0.0074
14	3	12	13	3	11	3830.1000	-0.0077
14	3	11	13	3	10	3830.2625	0.0026
14	2	12	13	2	11	3834.3750	0.0041
14	1	13	13	1	12	3849.9625	0.0054
12	1	11	11	0	11	3976.2000	0.0003
15	1	15	14	1	14	4077.4750	0.0146
15	0	15	14	0	14	4095.6375	0.0171
15	2	14	14	2	13	4101.7125	0.0009
15	5	10	14	5	9	4103.2375	-0.0242
15	4	11	14	4	10	4103.4375	-0.0078
15	3	13	14	3	12	4103.7750	0.0044
15	3	12	14	3	11	4103.9875	0.0016
15	2	13	14	2	12	4109.0125	0.0192
15	1	14	14	1	13	4124.6750	-0.0002
16	1	16	15	1	15	4349.0250	-0.0118
16	0	16	15	0	15	4367.6250	-0.0022
16	2	15	15	2	14	4374.9625	-0.0012
16	5	11	15	5	10	4376.8625	0.0169
16	4	12	15	4	11	4377.0750	0.0053
16	3	14	15	3	13	4377.4375	-0.0091
16	3	13	15	3	12	4377.7500	0.0056
16	2	14	15	2	13	4383.7500	-0.0011
16	1	15	15	1	14	4399.3375	0.0063
17	1	17	16	1	16	4620.5625	-0.0065
17	0	17	16	0	16	4639.4500	-0.0067
17	2	16	16	2	15	4648.1625	-0.0157
17	5	12	16	5	11	4650.4500	0.0141
17	4	13	16	4	12	4650.6875	-0.0184
17	3	15	16	3	14	4651.1375	0.0019
17	3	14	16	3	13	4651.5375	-0.0016
17	2	15	16	2	14	4658.6500	0.0040
17	1	16	16	1	15	4673.9125	-0.0069
18	1	18	17	1	17	4892.0500	-0.0053

18	0	18	17	0	17	4911.1000	-0.0070
18	2	17	17	2	16	4921.3500	-0.0028
18	5	14	17	5	13	4924.0500	0.0172
18	4	14	17	4	13	4924.3500	-0.0051
18	3	16	17	3	15	4924.8250	-0.0117
18	3	15	17	3	14	4925.3875	0.0133
18	2	16	17	2	15	4933.7000	0.0236
18	1	17	17	1	16	4948.4250	-0.0090
19	1	19	18	1	18	5163.5250	0.0301
19	0	19	18	0	18	5182.5750	-0.0038
19	2	18	18	2	17	5194.4875	0.0021
19	5	15	18	5	14	5197.6375	0.0005
19	4	15	18	4	14	5198.0125	-0.0057
19	3	17	18	3	16	5198.5250	-0.0243
19	3	16	18	3	15	5199.2500	-0.0035
19	2	17	18	2	16	5208.8500	0.0106
19	1	18	18	1	17	5222.8625	-0.0062
20	1	20	19	1	19	5434.9000	0.0133
20	0	20	19	0	19	5453.8750	0.0000
20	2	19	19	2	18	5467.6000	0.0266
20	5	15	19	5	14	5471.2500	0.0010
20	4	16	19	4	15	5471.6750	-0.0210
20	3	18	19	3	17	5472.2750	0.0027
20	3	17	19	3	16	5473.1875	0.0057
20	2	18	19	2	17	5484.1250	-0.0039
20	1	19	19	1	18	5497.2250	0.0079
21	1	21	20	1	20	5706.2375	0.0075
21	0	21	20	0	20	5724.9875	-0.0133
21	2	20	20	2	19	5740.5875	-0.0274
21	5	16	20	5	15	5744.8625	-0.0063
21	4	18	20	4	17	5745.3500	-0.0135
21	4	17	20	4	16	5745.3500	-0.0397
21	3	19	20	3	18	5746.0125	0.0083
21	3	18	20	3	17	5747.1750	0.0113
21	2	19	20	2	18	5759.5250	-0.0123
21	1	20	20	1	19	5771.4750	0.0027
22	1	22	21	1	21	5977.5375	0.0131
22	0	22	21	0	21	5995.9750	0.0111

Table S6. Rotational transitions of isomer 2 of the 2-adamantanol dimer (*Freq.*) and differences between observed and calculated transitions (*o-c*) for the fit of Table 3.

J'	K_{-1}'	K_{+1}'	J''	K_{-1}''	K_{+1}''	*Freq. / MHz*	*o-c / MHz*
8	1	8	7	1	7	2152.7250	-0.0057
8	0	8	7	0	7	2164.1125	0.0051
8	2	7	7	2	6	2164.9750	-0.0075
8	2	6	7	2	5	2166.0250	0.0180
8	1	7	7	1	6	2177.0500	0.0057
9	1	9	8	1	8	2421.7527	0.0086
9	0	9	8	0	8	2434.3000	-0.0083
9	2	8	8	2	7	2435.5500	-0.0005
9	3	6	8	3	5	2435.9625	-0.0188
9	2	7	8	2	6	2437.0125	0.0000
9	1	8	8	1	7	2449.1000	0.0087
10	1	10	9	1	9	2690.7250	-0.0056
10	0	10	9	0	9	2704.4000	-0.0003
10	2	9	9	2	8	2706.1000	0.0007
10	4	7	9	4	6	2706.5625	-0.0015
10	3	8	9	3	7	2706.6750	0.0042
10	3	7	9	3	6	2706.6750	-0.0200
10	2	8	9	2	7	2708.1000	-0.0067
10	1	9	9	1	8	2721.1000	-0.0079
11	1	11	10	1	10	2959.6875	-0.0005
11	0	11	10	0	10	2974.3750	0.0025
11	2	10	10	2	9	2976.6250	-0.0014
11	3	9	10	3	8	2977.4375	0.0515
11	3	8	10	3	7	2977.4375	0.0120
11	2	9	10	2	8	2979.3000	0.0017
11	1	10	10	1	9	2993.1000	0.0096
12	1	12	11	1	11	3228.6125	-0.0014
12	0	12	11	0	11	3244.2125	-0.0017
12	2	11	11	2	10	3247.1250	-0.0051
12	3	10	11	3	9	3248.1500	0.0365
12	3	9	11	3	8	3248.1500	-0.0248
12	2	10	11	2	9	3250.6000	0.0048
12	1	11	11	1	10	3265.0500	0.0154
13	1	13	12	1	12	3497.5000	-0.0061
13	0	13	12	0	12	3513.9250	0.0088
13	2	12	12	2	11	3517.6125	0.0045
13	4	9	12	4	8	3518.6500	0.0114
13	3	11	12	3	10	3518.9125	0.0588
13	3	10	12	3	9	3518.9125	-0.0330
13	2	11	12	2	10	3522.0125	0.0079

13	1	12	12	1	11	3536.9375	0.0013
14	1	14	13	1	13	3766.3500	-0.0125
14	0	14	13	0	13	3783.4750	0.0050
14	2	13	13	2	12	3788.0500	-0.0080
14	4	10	13	4	9	3789.3500	0.0025
14	3	12	13	3	11	3789.6000	-0.0067
14	3	11	13	3	10	3789.7375	-0.0029
14	2	12	13	2	11	3793.5250	-0.0077
14	1	13	13	1	12	3808.7875	-0.0035
15	1	15	14	1	14	4035.1875	0.0061
15	0	15	14	0	14	4052.8750	0.0068
15	2	14	14	2	13	4058.4750	-0.0031
15	4	12	14	4	11	4060.0875	0.0231
15	4	11	14	4	10	4060.0875	0.0210
15	3	13	14	3	12	4060.3500	-0.0229
15	3	12	14	3	11	4060.5500	-0.0122
15	2	13	14	2	12	4065.1750	-0.0095
15	1	14	14	1	13	4080.5875	-0.0069
16	1	16	15	1	15	4303.9500	-0.0108
16	0	16	15	0	15	4322.1000	-0.0050
16	2	15	15	2	14	4328.8625	-0.0035
16	4	13	15	4	12	4330.7875	-0.0053
16	4	12	15	4	11	4330.7875	-0.0086
16	3	13	15	3	12	4331.4000	-0.0139
16	2	14	15	2	13	4336.9500	-0.0135
16	1	15	15	1	14	4352.3375	-0.0039
17	1	17	16	1	16	4572.7125	0.0129
17	0	17	16	0	16	4591.1750	-0.0015
17	2	16	16	2	15	4599.2250	0.0050
17	5	13	16	5	12	4601.2875	0.0030
17	4	13	16	4	12	4601.5250	-0.0123
17	3	15	16	3	14	4601.9500	0.0059
17	3	14	16	3	13	4602.3250	0.0260
17	2	15	16	2	14	4608.8750	0.0035
18	1	18	17	1	17	4841.4000	0.0038
18	0	18	17	0	17	4860.0625	-0.0179
18	2	17	17	2	16	4869.5250	-0.0125
18	5	14	17	5	13	4871.9875	-0.0023
18	4	14	17	4	13	4872.2750	-0.0158
18	3	16	17	3	15	4872.7500	0.0018
18	3	15	17	3	14	4873.2250	0.0041
18	2	16	17	2	15	4880.9125	0.0044
19	1	19	18	1	18	5110.0625	0.0128
19	2	17	18	2	16	5153.0750	0.0038
20	2	19	19	2	18	5410.0875	0.0315
20	4	17	19	4	16	5413.8125	-0.0103

20	3	18	19	3	17	5414.4000	0.0096
20	3	17	19	3	16	5415.1750	-0.0155

Table S7. Comparison of the structural and energetic results for the two lowest-lying isomers of the 2-adamantanol dimer using B3LYP-D3(BJ), CAM-B3LYP-D3(BJ) and wB97XD (a def2-TZVP basis set as used in all cases). The experimental results correspond to the fit of Table 3.

	Experiment		Theory					
			B3LYP-D3(BJ)		CAM-B3LYP-D3(BJ)		ωB97XD	
	Isomer A	Isomer B	Isomer 1	Isomer 2	Isomer 1	Isomer 2	Isomer 1	Isomer 2
A / MHz [a]	701.117(17) [c]	702.79(27)	703.95[0.40%] [d]	702.74[-0.01%]	711.22[1.44%]	709.01[0.89%]	700.34[-0.11]	697.27[-0.79%]
B / MHz	138.34738(18)	136.84142(21)	140.03[1.22%]	139.42[1.88%]	139.85[1.09%]	141.61[3.49%]	144.51[4.46]	146.28[6.90%]
C / MHz	135.18537(18)	133.80126(23)	136.84[1.22%]	136.13[1.74%]	136.776[1.18%]	138.42[3.45%]	140.97[4.48%]	142.52[6.52%]
κ	-0.9888(4)	-0.9893(8)	-0.9890	-0.9880	-0.9893	-0.9888	-0.9873	-0.9864
D_J / kHz	0.00643(18)	0.00471(23)	0.0063	0.0045	0.0068	0.0066	0.0038	0.0037
D_{JK} / kHz	-0.0169(54)		-0.0150	0.0142	-0.0192	-0.0115	-0.0060	-0.0029
D_K / kHz			0.0532	0.0104	0.0605	0.0438	0.0300	0.0237
d_1 / kHz			-0.0003	-0.0002	-0.0004	-0.0004	-0.0002	-0.0002
d_2 / kHz			0.0000	0.0000	0.000	0.0000	0.0000	0.0000
$\|\mu_a\|$ / D			3.12	2.66	3.16	2.66	3.09	2.50
$\|\mu_b\|$ / D			0.23	0.31	0.22	0.68	0.24	0.77
$\|\mu_c\|$ / D			1.10	1.21	1.15	0.85	1.19	0.77
ΔE_{ZPE} / kJ mol^{-1} [b]			0.00	0.00	0.00	1.75	0.00	0.59
$\Delta G_{298\,K}$ / kJ mol^{-1}			0.97	0.00	0.00	1.94	0.00	0.12

[a]Rotational constants (*A*, *B*, *C*), Ray's asymmetry parameter (κ = (2*B*-*A*-*C*)/(*A*-*C*)), Watson's S-reduction centrifugal distortion constants (D_J, D_{JK}, D_K, d_1, d_2) and electric dipole moments (μ_α, α = a, b, c). [b]Relative electronic energy with zero-point correction (ΔE_{ZPE}) and Gibbs energy (Δ*G*, 298K, 1 atm). [c]Standard errors in parentheses in units of the last digit. [d]Relative differences with the experimental rotational constants.

Table S8. Relative intensity measurements for a set of ^{a}R rotational transitions of isomers 1 and 2 of the 2-adamantanol dimer. The population ratio was calculated with the B3LYP-D3(BJ) electric dipole moments of Table 3.

J'	K_{-1}'	K_{+1}'	J''	K_{-1}''	K_{+1}''	Intensity isomer 1 / a.u.	Intensity isomer 2 / a.u.	Population Ratio 1 : 2
10	0	10	9	0	9	0.000246	0.000086	2.44
10	1	10	9	1	9	0.000245	0.000068	3.08
10	1	9	9	1	8	0.000233	0.000081	2.44
9	0	9	8	0	8	0.000285	0.000071	3.45
11	0	11	10	0	10	0.000338	0.000091	3.17
11	1	11	10	1	10	0.000238	0.000106	1.91
11	1	10	10	1	9	0.000331	0.000080	3.53
12	0	12	11	0	11	0.000219	0.000095	1.97
13	0	13	12	0	12	0.000245	0.000105	1.99
13	1	13	12	1	12	0.000293	0.000101	2.47
13	1	12	12	1	11	0.000347	0.000096	3.08
							Average	2.3
							Standard deviation	0.5

Table S9. Atomic coordinates for isomer 1 of the 2-adamantanol dimer in the principal inertial axes system (B3LYP-D3(BJ)/def2-TZVP).

	Atom	*a*	*b*	*c*
1	O	1.2283	1.6767	-0.6945
2	C	2.0643	0.0972	0.9119
3	C	3.5987	1.0205	-0.8426
4	C	4.4994	-0.5242	0.9182
5	C	2.9800	-1.4104	-0.8746
6	C	2.3991	1.3248	0.0632
7	C	3.2783	-0.2211	1.7981
8	C	1.7599	-1.1053	0.0060
9	C	4.8097	0.7029	0.0487
10	C	3.2874	-0.1826	-1.7443
11	C	4.1917	-1.7268	0.0138
12	H	1.1988	0.3304	1.5351
13	H	3.8156	1.9026	-1.4558
14	H	5.3611	-0.7494	1.5519
15	H	2.7620	-2.2670	-1.5176
16	H	2.6483	2.1605	0.7282
17	H	3.4924	0.6205	2.4632
18	H	3.0485	-1.0799	2.4342
19	H	1.5135	-1.9720	0.6257
20	H	0.8902	-0.8980	-0.6178
21	H	5.0489	1.5618	0.6827
22	H	5.6861	0.5128	-0.5766
23	H	2.4355	0.0412	-2.3891
24	H	4.1438	-0.3829	-2.3943
25	H	3.9871	-2.6106	0.6251
26	H	5.0619	-1.9604	-0.6069
27	H	1.4247	2.4521	-1.2315
28	O	-1.2176	1.6185	0.7946
29	C	-3.0085	0.1564	1.4330
30	C	-2.0136	-0.0246	-0.8599
31	C	-3.7810	-1.6499	-0.1318
32	C	-4.3832	0.7481	-0.5814
33	C	-1.7227	0.3035	0.6147
34	C	-3.4920	-1.2977	1.3348
35	C	-4.0902	1.0981	0.8850
36	C	-2.4951	-1.4795	-0.9538
37	C	-3.0966	0.9177	-1.4022
38	C	-4.8641	-0.7074	-0.6779

39	H	-2.7851	0.4110	2.4720
40	H	-1.0912	0.1008	-1.4343
41	H	-4.1255	-2.6856	-0.1997
42	H	-5.1569	1.4159	-0.9705
43	H	-0.9904	-0.4246	0.9918
44	H	-2.7357	-1.9764	1.7409
45	H	-4.3954	-1.4281	1.9376
46	H	-5.0003	1.0000	1.4846
47	H	-3.7533	2.1325	0.9660
48	H	-1.7203	-2.1577	-0.5827
49	H	-2.6778	-1.7444	-1.9995
50	H	-2.7506	1.9513	-1.3450
51	H	-3.2874	0.6927	-2.4561
52	H	-5.7904	-0.8347	-0.1092
53	H	-5.0891	-0.9604	-1.7187
54	H	-0.4036	1.7054	0.2712

Table S10. Atomic coordinates for isomer 2 of the 2-adamantanol dimer in the principal inertial axes system (B3LYP-D3(BJ)/def2-TZVP).

	Atom	*a*	*b*	*c*
1	O	-1.2680	1.7273	0.6524
2	C	-1.9542	-0.0847	-0.7731
3	C	-3.6630	1.1481	0.5861
4	C	-4.3922	-0.6489	-1.0065
5	C	-3.1564	-1.2554	1.0944
6	C	-2.3454	1.2731	-0.1880
7	C	-3.0557	-0.5205	-1.7514
8	C	-1.8213	-1.1257	0.3479
9	C	-4.7609	0.7112	-0.3964
10	C	-3.5221	0.1057	1.7046
11	C	-4.2550	-1.6905	0.1140
12	H	-1.0052	0.0239	-1.3009
13	H	-3.9202	2.1242	1.0133
14	H	-5.1735	-0.9587	-1.7053
15	H	-3.0602	-1.9977	1.8909
16	H	-2.4723	1.9908	-1.0075
17	H	-3.1464	0.2035	-2.5665
18	H	-2.7819	-1.4772	-2.2036
19	H	-1.5329	-2.0876	-0.0843
20	H	-1.0301	-0.8349	1.0392
21	H	-4.8801	1.4579	-1.1872
22	H	-5.7179	0.6449	0.1281
23	H	-2.7519	0.4161	2.4132
24	H	-4.4632	0.0336	2.2572
25	H	-4.0100	-2.6674	-0.3130
26	H	-5.2076	-1.8004	0.6410
27	H	-1.5107	2.5798	1.0306
28	O	1.2112	1.6093	-0.7686
29	C	2.0043	-0.0846	0.8370
30	C	3.5584	1.1265	-0.7098
31	C	4.4787	-0.4783	0.9891
32	C	3.1853	-1.3409	-0.9839
33	C	2.2685	1.2441	0.1073
34	C	3.1744	-0.3656	1.7914
35	C	1.8822	-1.2270	-0.1800
36	C	4.7271	0.8417	0.2447
37	C	3.4320	-0.0185	-1.7251
38	C	4.3544	-1.6219	-0.0284

39	H	1.0760	0.0094	1.4075
40	H	3.7202	2.0717	-1.2338
41	H	5.3115	-0.6789	1.6690
42	H	3.0993	-2.1564	-1.7074
43	H	2.4055	2.0323	0.8622
44	H	3.2584	0.4359	2.5319
45	H	2.9892	-1.2920	2.3430
46	H	1.6745	-2.1657	0.3426
47	H	1.0456	-1.0383	-0.8538
48	H	4.8387	1.6608	0.9617
49	H	5.6608	0.7837	-0.3221
50	H	2.6103	0.1877	-2.4125
51	H	4.3488	-0.0850	-2.3188
52	H	4.1933	-2.5704	0.4934
53	H	5.2859	-1.7209	-0.5943
54	H	0.3938	1.6923	-0.2494
